# Radiotherapy as a primary treatment modality for a young man with a primary urethral plasmacytoma: case report and literature review

**DOI:** 10.3389/fonc.2025.1528536

**Published:** 2025-02-13

**Authors:** Soo How Lim, Tian Er Poh, Hong Chin Wee, Nur Shazwaniza Binti Awang Basry, Mohd Syazwan Bin Tajul Arifin, Kar Ying Yong

**Affiliations:** ^1^ Clinical Research Centre, Miri Hospital, Miri, Malaysia; ^2^ Institute of Clinical Research, National Institute of Health, Selangor, Malaysia; ^3^ Clinical Research Centre, Penang Hospital, Penang, Malaysia; ^4^ Pathology Department, Sarawak General Hospital, Kuching, Malaysia; ^5^ Surgical Department, Miri Hospital, Miri, Malaysia; ^6^ Medical Department, Miri Hospital, Miri, Malaysia

**Keywords:** plasmacytoma, urethral, treatment modality, radiotherapy, preserve function, case report

## Abstract

Primary urethral plasmacytoma is an extremely rare form of solitary plasmacytoma, with only 10 cases reported in the literature. It involves localized clonal proliferation of plasma cells without systemic disease. This report presents a 29-year-old man with acute urinary retention and a urethral mass, confirmed as solitary plasmacytoma. The patient was treated with 45 Gy of local radiotherapy, resulting in complete tumor resolution without recurrence or progression at a 2-year follow-up. Given its rarity, treatment strategies for primary urethral plasmacytoma are not well-defined. Radiotherapy is preferred over surgery in young patients due to the radiosensitivity of plasma cell tumors and its ability to preserve sexual and urinary function. A review of previous cases treated with radiotherapy alone, using doses of 40–50 Gy, showed favorable outcomes with no recurrences reported over follow-up periods ranging from 6 months to 12 years. Only one patient experienced minor long-term complications. This report highlights the effectiveness of radiotherapy as a primary modality for managing primary urethral plasmacytoma, offering excellent local control while preserving organ function. Individualized treatment plans should consider patient age, fertility concerns, and tumor characteristics. Further research is necessary to optimize treatment protocols and long-term surveillance strategies due to the potential risk of recurrence or progression to multiple myeloma.

## Introduction

Solitary plasmacytoma (SP) is a localized neoplasm characterized by monoclonal proliferation of the B cells without systemic involvement, which can be further divided into solitary bone plasmacytoma and solitary extramedullary plasmacytoma. Solitary extramedullary plasmacytoma predominantly involves the soft tissues with the aerodigestive tract being the most frequently affected site (1). Solitary extramedullary plasmacytoma of the urethra is extremely rare. To date, there have been just 10 published reports of primary urethral plasmacytoma (PUP) (**Table 1**). We reported a case of PUP of a young gentleman who was successfully treated with radiotherapy alone with no evidence of relapse in 2 years and with no short-term and long-term radiation adverse effects. We aim to elucidate the treatment modalities of PUP and to provide a more refined approach to its management in clinical practice.

**Table 1:** Solitary plasmacytoma of the genitourinary tract—review of the literature.

Author	Diagnosis	Clinical history	Treatment modality	Follow-up period	Outcome
Mordkin et al. (5)	Plasmacytoma of the urethra	39-year-old man presented with 15-month history of dysuria, frequency, and penile pruritus	External beam radiotherapy (total dose of 41.4 Gy)	>12 years	No recurrent or metastasis No significant sequelae
Gokce et al. (6)	Primary urethral plasmacytoma	51-year-old man presented with terminal hematuria, palpable penile mass, and mild dysuria	External beam radiotherapy (total dose of 40 Gy) targeting the perineal region	6 months	No recurrent or metastasis No mention of radiation effect
Alcorn et al. (7)	Solitary plasmacytoma of the penile urethra	35-year-old man presented with 2 months of painless hematuria	Radiotherapy - Total dose of 50.4 Gy (45 Gy in 1.8 Gy/fraction to penile urethra and inguinal lymph nodes, 5.4 Gy to penile urethra)	1 year	No recurrence or metastasis No long-term radiation effect
Stein et al. (8)	Primary urethral solitary plasmacytoma	22-year-old man presented with a palpable induration of the ventral penis, whitish urethral discharge in the morning, intermittent dysuria, hematospermia, and pain during coitus	Localized high-dose brachytherapy - Total dose of 43 Gy, 14 fractions over 19 days	15 months	Intermittent transurethral bleeding post-coitus, induration of the ventral penis still palpable, otherwise disease-free
Witjes et al. (9)	Extramedullary plasmacytoma of the urethra	39-year-old man presented with blood-stained clothing, dysuria, and swollen distal portion of penis	1st: external radiotherapy (total dose of 46 Gy in 23 sessions) 2nd: urethrectomy and perineostomy	1 year	Relapse 1 year post-initial therapy, no recurrent or metastasis after 2nd treatment modal
Campbell et al. (10)	Plasmacytoma of the urethra	73-year-old man presented with hematuria and a mass in the distal urethra. Patient had history of solitary plasmacytoma of the oral pharynx 7 years ago, which was treated with surgical excision and postoperative irradiation	Distal urethrectomy, preserving the glans penis	1 year	No recurrence or metastasis
Kraus et al. (11)	Plasmacytoma of the urethra	35-year-old woman presented with 1-year history of hesitancy in voiding	1st: urethrotomy 2nd: urethrotomy and external beam radiotherapy (total dose of 45 Gy with fractionation of 1.8 Gy per 5 days a week)	3 years	Local recurrence 3 months later after first urethrotomy. No recurrent or metastasis after second urethrotomy and radiotherapy
Mark et al. (12)	Plasmacytoma of the urethra	23-year-old woman presented with urethral bleeding post-wiping, a decreased caliber of urinary stream, and hesitancy. History of diethylstilbestrol exposure	External meatus resection + external beam radiotherapy (20 treatments for a total dose of 36 Gy)	>10 years	No recurrent or metastasis
Su et al. (13)	Plasmacytoma of external urethral meatus	50-year-old woman presented with painless gross hematuria	Urethral meatus lumpectomy combined with radiotherapy [Planning target volume (PTV) D95% = 43.2 Gy/24 fractions; planning target gross tumor volume (PTGV) D95% = 48 Gy/24]	1 year	No recurrence or metastasis
Lemos et al. (14)	Solitary plasmacytoma of the urethra	56-year-old woman presented with dysuria accompanied by urethral bleeding (when wiping her genital area with toilet paper)	Surgical excision	3 years	No recurrence or metastasis

## Case presentation

**Figure 1:**
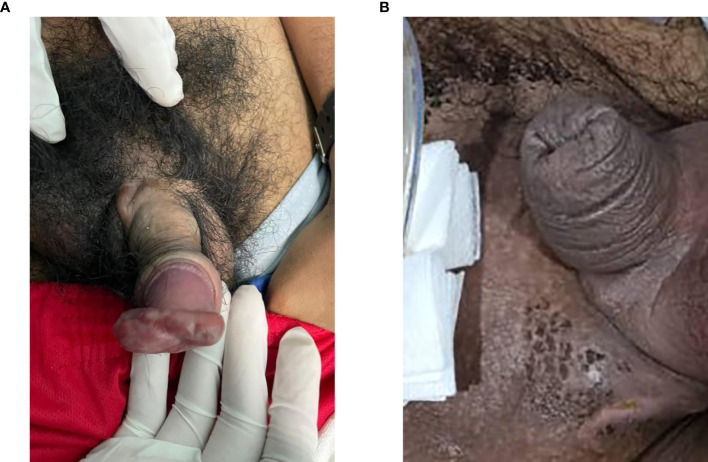
**(A)** Mass of fungating tumor at the tip of the penile glans measuring 2 × 3 cm. **(B)** Resolution of the penile mass.

**Figure 2:**
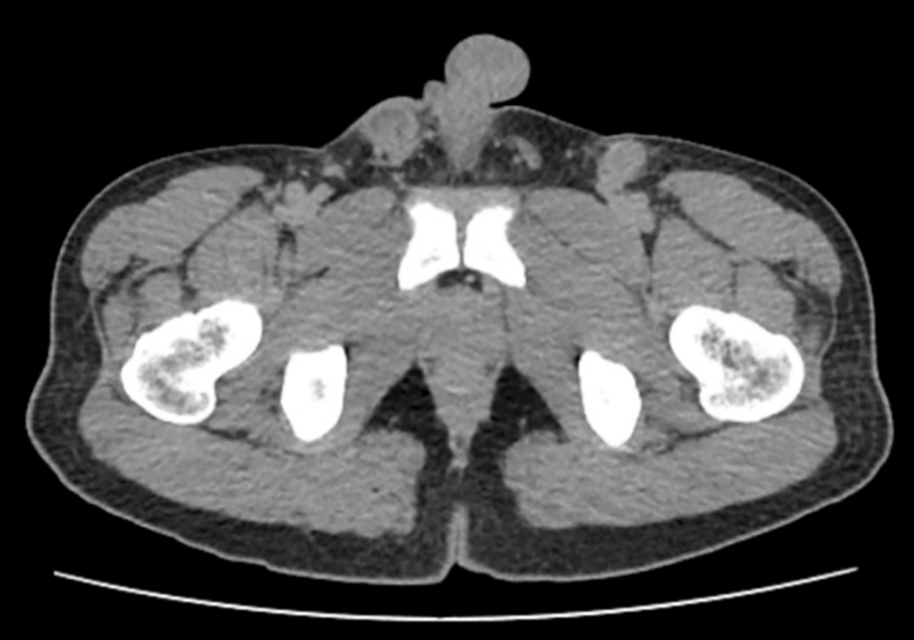
Contrast-enhanced computed tomography (CECT) pelvis in axial view showed enlarged iliac lymph node and inguinal lymph nodes with necrotic center.

**Figure 3:**
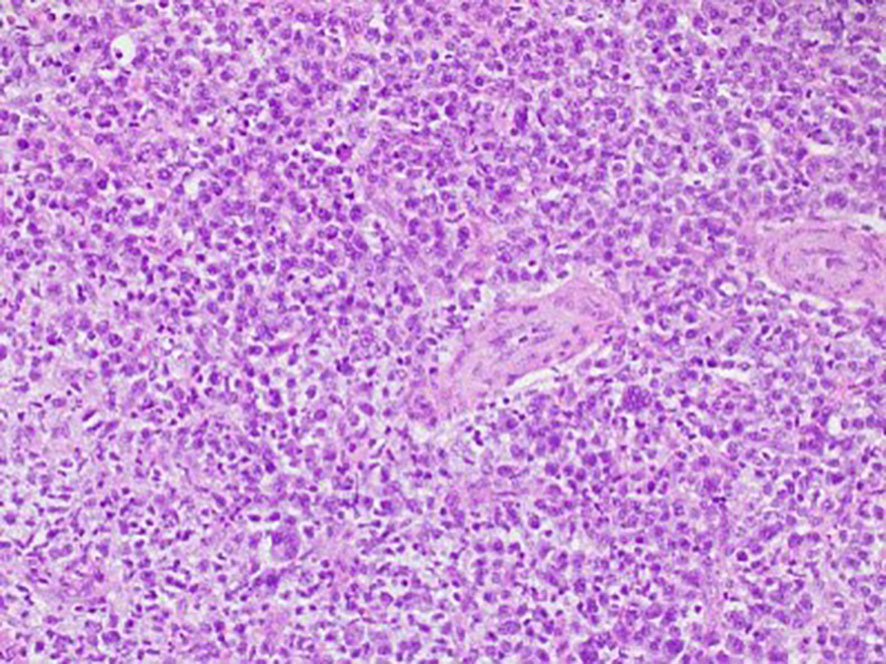
Histology of the glans penis. Neoplastic cells with a plasmacytoid appearance, including binucleated and multinucleated forms, are present.

A 29-year-old married man, with no known medical history, presented with acute urinary retention requiring urgent suprapubic catheter (SPC) insertion. He reported a 2-month history of painless growth on the penis. On physical examination, a fungating mass, measuring 2 × 3 cm, was observed at the tip of the glans (**Figure 1A**). A wedge biopsy of the glans revealed florid granulomatous inflammation with atypical plasma cell infiltration with predominance of lambda light chain expression suggestive of plasma cell neoplasm (PCN). However, bone marrow examination ruled out plasma cell neoplasm, and the patient had no end-organ damage such as hypercalcemia, renal impairment, anemia, or bone lesions. He had no detectable paraprotein too. Contrast-enhanced computed tomography (CECT) of the neck, thorax, abdomen, and pelvis revealed bilateral inguinal lymphadenopathy, with the largest node on the right inguinal side measuring 2.4 × 2.0 × 2.8 cm, and bilateral iliac lymphadenopathy, with the largest node on the right external iliac side measuring 1.6 × 3.0 cm (**Figure 2**) . Excisional biopsies of the bilateral inguinal lymph nodes showed no malignancy. Diagnostic antegrade flexible cystoscopy revealed meatal stenosis and a distal urethral mass extending to the base of the penis. Histology showed a neoplastic cells with a plasmacytic appearance, including binucleated and multinucleated forms (**Figure 3**).

The patient was diagnosed with PUP. After a multidisciplinary team discussion involving a urologist, an oncologist, and a hematologist, the patient underwent local definitive radiotherapy. A total dose of 45 Gy was delivered in 25 fractions over 5 weeks, targeting the penis and right inguinal lymph nodes. The treatment utilized 6-MV photons, ensuring 95% coverage of the target volume. The radiotherapy was administered using a three-dimensional external beam radiation therapy (3D-EBRT) technique with anterior and posterior fields. One month post-radiotherapy, the penile mass had resolved, with no urinary or sexual dysfunction (**Figure 1B**). The SPC was removed via flexible cystoscopy. At the 2-year follow-up, the patient remained well, with no long-term radiation side effects and no evidence of progression to multiple myeloma.

## Discussion

The primary treatment modalities for PUP include surgery, radiotherapy, or a combination of both. However, managing young men with PUP poses distinct challenges for treating physicians, especially in relation to fertility preservation and maintaining erectile function. Given these concerns, radiotherapy is often the preferred treatment, especially for patients of reproductive age. Plasma cell tumors are radiosensitive, and radiotherapy has been associated with favorable survival outcomes (2–4). Four case reports have documented successful treatment of PUP in men using radiotherapy alone, with doses ranging from 40 to 50 Gy (5–8). In these cases, no recurrences were reported, with follow-up periods ranging from 6 months to 12 years. All have no reported long-term adverse events except in the study by Stein et al., who reported that one patient experienced intermittent transurethral bleeding post-coitus. These cases highlight the efficacy of radiotherapy in providing local tumor control while preserving organ function. However, Witjes et al. reported a 39-year-old gentleman with PUP who relapsed after 1 year of 46-Gy external radiotherapy but achieved treatment remission following urethrectomy and perineostomy (9).

Surgical resection alone has been less commonly reported. Campbell et al. described a 73-year-old man with distal urethral plasmacytoma who had a prior history of solitary plasmacytoma of the oropharynx treated with surgical excision and radiotherapy 7 years earlier. He underwent distal urethrectomy with preservation of the glans penis and remained in remission for 1 year (10). However, Kraus et al. reported a 35-year-old gentleman with PUP who had local recurrence after 3 months of initial urethrotomy but achieved disease remission after a second urethrotomy and 46 Gy of radiotherapy (11).

There are also three reported cases of PUP in women (12–14). The first two cases by Mark et al. and Su et al. were treated successfully with combined surgery and radiotherapy, with follow-up ranging from 1 year to more than 10 years (9, 10). Lemos et al. reported a case of a woman who responded well to surgical resection alone, with no evidence of relapse after 3 years of follow-up (14).

In conclusion, case reports have shown that radiotherapy alone can achieve favorable outcomes in tumor control while preserving the integrity of the sexual organ, enabling men of reproductive age to maintain their sense of masculinity without experiencing psychological or physical burdens. However, treatment strategies for patients with PUP should be personalized, as individual responses may vary depending on the underlying disease biology. Therefore, if the initial treatment approach fails or proves ineffective, a second alternative or combined treatment modality should be considered. Long-term follow-up and surveillance are crucial to detect any potential relapse or progression to multiple myeloma (15).

## Patient’s perspective

“I was really worried at first about the problems I was facing. Before starting treatment, I was told there could be many potential side effects from radiotherapy. But thankfully, I didn’t experience many issues. Now, I’m back at work and feeling so happy with the results.”

## Data Availability

The raw data supporting the conclusions of this article will be made available by the authors, without undue reservation.
